# Radiological Evaluation of Vascular Structures in Cats Infected with Immature Worms of *Dirofilaria immitis*

**DOI:** 10.3390/ani14202943

**Published:** 2024-10-11

**Authors:** Soraya Falcón-Cordón, Yaiza Falcón-Cordón, Sara Nieves García-Rodríguez, Noelia Costa-Rodríguez, Daniel Julio Vera-Rodríguez, José Alberto Montoya-Alonso, Elena Carretón

**Affiliations:** Internal Medicine, Veterinary Medicine and Therapeutic Research Group, Faculty of Veterinary Medicine, Research Institute of Biomedical and Health Sciences (IUIBS), Universidad de Las Palmas de Gran Canaria (ULPGC), 35016 Las Palmas de Gran Canaria, Spain; soraya.falcon@ulpgc.es (S.F.-C.); yaiza.falcon@ulpgc.es (Y.F.-C.); saranieves.garcia@ulpgc.es (S.N.G.-R.); noelia.costa@ulpgc.es (N.C.-R.); daniel.vera103@alu.ulpgc.es (D.J.V.-R.); elena.carreton@ulpgc.es (E.C.)

**Keywords:** feline heartworm disease, imaging diagnosis, thoracic radiography, vector-borne disease, radiographic indexes, cardiac silhouette, vascular enlargement

## Abstract

**Simple Summary:**

This study aimed to identify thoracic radiographic abnormalities in cats infected with immature worms of *Dirofilaria immitis*. A total of 123 cats from a hyperendemic area were included and divided into healthy cats (n = 50), asymptomatic cats who were seropositive to *D. immitis* antibodies (n = 30), and seropositive cats with clinical signs (n = 43). Different radiographic measurements were assessed including the VHS and CrPA/R4, CdPA/R9, CVC/Ao, and CVC/R4 ratios. The results showed that significant differences were observed between healthy and infected cats for all except the VHS, demonstrating enlarged vasculature in cats with *D. immitis*. Moreover, 62.8% of the cats with clinical signs showed a marked bronchointerstitial pattern, while asymptomatic cats mainly (33.3%) had a mild bronchointerstitial pattern. This study highlights the importance of thoracic radiography in diagnosing and monitoring heartworm disease in cats.

**Abstract:**

This study aimed to assess thoracic radiographic abnormalities in cats infected with immature stages of *Dirofilaria immitis* to evaluate the utility of this diagnostic technique during early infection. A total of 123 cats from a hyperendemic area were classified into three groups: asymptomatic cats seronegative to anti-*D.-immitis* antibodies (Group A), seropositive asymptomatic cats (Group B), and seropositive cats with clinical signs that were at high risk of heartworm-associated respiratory disease (HARD) (Group C). Radiographic measurements and lung parenchymal abnormalities were analyzed and compared across the groups. Significant differences in several parameters, including CrPA/R4, and CdPA/R9 ratios, were observed between healthy and seropositive cats, suggesting early arterial damage even in the absence of adult worms. Other parameters that showed differences between healthy and infected cats were CVC/Ao and CVC/R4 ratios, but not the VHS. Group C exhibited a marked bronchointerstitial pattern, indicating severe parenchymal alterations associated with clinical signs. The study demonstrated that thoracic radiography can detect early vascular and parenchymal changes in feline *D. immitis* infections, providing valuable information for diagnosing HARD. However, it also highlights the limitations of radiographic techniques, as some seropositive cats displayed no significant abnormalities. The findings underscore the importance of combining radiography with clinical and serological assessments for a more accurate diagnosis of feline heartworm disease.

## 1. Introduction

*Dirofilaria immitis* is a nematode parasite that causes heartworm disease. It has a cosmopolitan distribution and is considered endemic in the Canary Islands [1,2]. While cats can become infected, they are more resilient to infections with adult *D. immitis* worms compared to dogs [3]. The pathophysiology of feline heartworm is basically differentiated into two stages; the first stage is associated with the arrival of immature heartworms in the pulmonary vasculature, and the second stage is related to the presence and death of adult worms [4,5,6].

The first stage happens approximately 3–4 months post-infection, with the arrival of immature worms in the pulmonary arteries and arterioles and subsequent death of most of them, mainly due to the action of the intravascular alveolar macrophages. This reaction causes clinical signs due to an acute vascular and parenchymal inflammatory response [6,7]. These signs are mostly respiratory in nature and this symptomatic phase is referred to as heartworm-associated respiratory disease (HARD) [8,9]. Those larvae that manage to develop and reach adulthood cause the second stage of the disease. In general, cats have a low parasite burden and the longevity of the worms is relatively short [10].

Given the complicated diagnosis of feline heartworm, a combination of serological and imaging techniques is usually recommended to detect adult parasites. Feline heartworm disease is a dynamic disease, and all the diagnostic tests carried out should be performed and studied altogether to determine whether the animal is indeed infected by adult parasites or whether there is a high index of suspicion of infection [11,12,13].

Among the diagnostic techniques available, thoracic radiography is widely used for evaluating the pulmonary parenchyma and vascular structures in feline cardiopulmonary diseases [14]. In *D. immitis* infections, an enlargement of the main and peripheral pulmonary arteries has been described, characterized by the loss of the conical shape, tortuosity, and truncation of the caudal lobar branches. Additionally, parenchymal alterations are commonly observed, with diffuse or focal bronchointerstitial patterns detectable on radiographs [10,15,16,17]. However, these lesions are not patognomonic and are similar to the lesions found in other diseases, such as infections by *Toxocara cati* or *Aerostrongylus* spp., asthma, or allergic bronchitis [10,18,19,20]. In most cases, these alterations are accompanied by clinical signs such as coughing or dyspnea, among others [5,10,16]. In any case, a radiological study, used in combination with the clinical history, has proven essential for establishing a correct diagnosis [21,22].

Regarding studies related to HARD induced by immature *D. immitis* in cats, it has been reported that histopathological lesions were mainly focused on the main pulmonary artery and arterioles; this caused bronchointerstitial changes, such as bronchiolar lesions and partial obstruction of some primary bronchi, hyperplasia and hypertrophy of the muscular layer and medial hypertrophy of the small pulmonary arterioles, and interstitial lung disease [6,9,10,23]. Moreover, the studies reported that these alterations may result in pulmonary endarteritis, indicating that even transient infection can cause long-term lesions in cats [10,23] that may be detectable with thoracic radiography, which is the aim of the present study. However, other studies reported that the minor distinctions observed between infected and healthy cats indicated that clinical use of thoracic radiology was very limited or, in some cases, thoracic radiographs provided no evidence of infection in cats with immature infections [10,17]. Therefore, the aim of this study was to identify thoracic radiographic abnormalities in cats infected with immature stages of *D. immitis* to determine the utility of this diagnostic technique at this stage of infection.

## 2. Materials and Methods

### 2.1. Study Animals

A total of 123 rescued and client-owned cats brought to the Veterinary Teaching Hospital of the University of Las Palmas de Gran Canaria were included in the study. These cats lived in a hyperendemic area for *D. immitis* [1,2]. They were cats that participated in a feline heartworm screening campaign for cats over 7 months of age who never received heartworm chemoprophylaxis. Clinical history and data were recorded for each animal, including age, sex, and breed. All owners provided their consent for participation in this study. The study was conducted in accordance with current European legislation on animal protection.

The cats were further examined for the presence or absence of clinical signs related to feline heartworm, such as coughing, dyspnea, tachypnea, increased respiratory effort, vomiting, systolic heart murmur, anorexia and weight loss, ascites, syncope, or neurological signs.

In addition, each feline patient underwent a thorough medical history and physical examination to eliminate the possibility of other conditions that could impact the results; cats with concomitant diseases were not included in the study. Additionally, testing for feline immunodeficiency virus (FIV) and feline leukemia virus (FeLV) was also carried out and any cat that tested positive was excluded from the study.

Out of the 123 cats surveyed, 42.35% (52/123) were male while 57.65% (68/123) were female. There were 170 European Shorthair cats, 2 Sphynx cats, 1 Maine Coon cat, and 1 Turkish Angora cat included in the breed categorization.

### 2.2. Serology

Blood samples were taken from either the cephalic or jugular vein and spun in dry tubes to check their serological status. The serum was stored at a temperature of −20 °C until the tests were conducted. The presence of feline *D. immitis* infection was determined through serological methods to detect specific antibodies against *D. immitis* using an indirect enzyme-linked immunosorbent assay (ELISA) (in-house ELISA, Urano Vet^®^, Barcelona, Spain). In short, each well of the ELISA plate was covered with recombinant *D. immitis* antigens (Di33 protein, 0.5 ug/mL). The sample diluent was mixed with the serum in a 1:100 dilution ratio. Following an initial wash to eliminate unbound molecules, the TMB substrate, labeled with horseradish peroxidase, was introduced, targeting feline IgG specifically. The readings of absorbance (or optical density) were taken at 450 nm within a 5-min time frame following the introduction of the stop solution (sulfuric acid). As per the kit manufacturer’s guidelines, seronegativity was defined at a cut-off of < 1, while seropositivity was defined at a cut-off of 1 or higher. Furthermore, all samples were tested for circulating *D. immitis* antigens using a commercial immunochromatographic test kit (Uranotest Dirofilaria ©, UranoVet SL, Barcelona, Spain) according to the manufacturer’s instructions.

### 2.3. Imaging Diagnosis

Echocardiograms were conducted on every cat to confirm the presence or absence of adult worms, as well as to exclude any other concurrent diseases. The cats were positioned on their right side with the transducer positioned in the third intercostal space to check for worms in the pulmonary arteries and right-sided heart chambers. Cats remained conscious and were continuously monitored with electrocardiography throughout the entire test.

**Figure 1:**
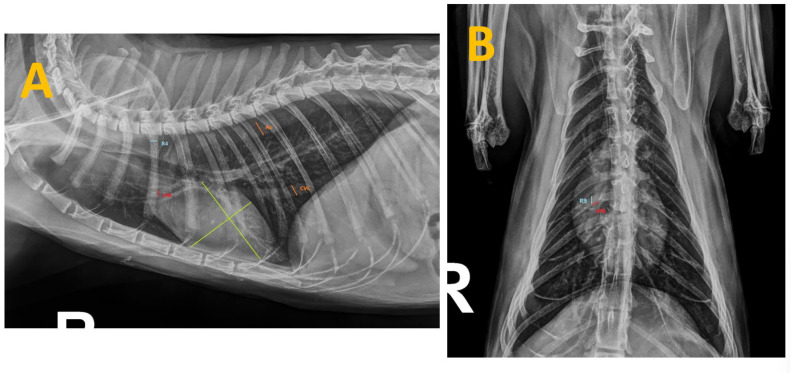
Thoracic radiographs of a cat seropositive for anti-Dirofilaria-immitis antibodies and presenting with clinical signs. The measurements taken during this study are shown as follows: (**A**) Right laterolateral projection illustrating the measurements of the caudal vena cava (CVC) and aorta (Ao) (in orange), the fourth rib (R4) (in blue), the right cranial pulmonary artery (CrPA) (in red) and the vertebral heart score (in green). (**B**) Dorsoventral projection displaying the measurement of the right caudal pulmonary artery (CdPA) (in red) in relation to the ninth rib (R9) (in blue).

Thoracic radiographs from all cats were taken using the same radiographic equipment (Bennett HFQ-600P, Greensboro, NC, USA) during inspiration and without sedation to minimize changes in heart size [24]. Views were obtained in both right laterolateral and dorsoventral projections, and radiographic measurements were taken using adjustable calipers by an unbiased operator (SFC), who has 10 years of clinical experience in cardiorespiratory diseases in small animals and was blinded to the clinical status of the study cats (Figure 1).

In lateral recumbent radiographs, the Vertebral Heart Score (VHS) measurement was obtained from the sum of the short-axis and the long-axis measurements as previously described [25]. The cardiac long axis was obtained from the cardiac apex to the base of the heart where it meets the trachea just cranial to the carina, expressed as the number of vertebral lengths in the lateral radiograph, measured caudally from the cranial border of T4. The short axis of the heart was measured perpendicular to the long-axis measurement at the point of maximum heart width, expressed as the number of vertebral lengths in the lateral radiograph, measured caudally from the cranial border of T4 [26].

In the laterolateral projection, the diameter of the right fourth rib (R4) just below the spine and the greatest diameter of the caudal vena cava (CVC) were also measured as described in previous studies, which included dogs with heartworm, in order to determine the mean caudal vena cava size, expressed as a ratio of the diameter of R4 [27,28,29]. The measurement of the diameter of the descending aorta (Ao) at the same intercostal space as the CVC was carried out as well, following previous guidelines in dogs [29]. Next, the CVC/Ao and CVC/R4 ratios were established.

Other radiological measurements were taken, including the diameter of the right cranial pulmonary artery (CrPA) passing through R4 in the laterolateral projection and the diameter of R4 at a point just distal to the spine. Moreover, in the dorsoventral projections, the distal and left sides of the summation shadow created by the right caudal pulmonary artery (CdPA) with R9 were measured. Finally, the CrPA/R4 and CdPA/R9 ratios were calculated from these measurements [30].

In addition, quantitative evaluation of the shape and tortuosity of the lung vasculature in both radiographic projections took place. Finally, the parenchyma of all radiographs was examined to determine the presence or absence of radiological abnormalities and their nature, classified as bronchial pattern, vascular pattern, interstitial pattern, alveolar pattern, or mixed patterns in all groups.

### 2.4. Statistical Analyses

The data were analyzed using SPSS Base 29.0 software for Windows. A Shapiro–Wilk test was performed to verify the normal distribution of the data. Additionally, a Siegel–Tukey test was performed to verify the variability of variances between groups. The chi-squared test was used to assess the association between categorical variables. A non-parametric Mann–Whitney U test was performed to determine differences between the groups for all recruited-cat measurements. In all cases, a *p*-value < 0.05 was determined as significant. Continuous variables were expressed as the median ± standard deviation, while qualitative variables were expressed as percentages. In all cases, a *p*-value < 0.05 was considered significant. In addition, Pearson’s correlation coefficient was obtained to determine the relationship between variables. The strength of the correlations was categorized according to standard conventions: a correlation coefficient of r ≤ 0.30 was considered weak, 0.31 ≤ r ≤ 0.50 was classified as moderate, and r > 0.50 was regarded as strong.

## 3. Results

Based on the results, cats were divided into three groups: Group A (n = 50) consisted of cats with no clinical signs that were seronegative for anti-*D.-immitis* antibodies, Group B (n = 30) consisted of cats seropositive to *D. immitis* but who were asymptomatic, and Group C (n = 43) comprised seropositive animals with *D. immitis* exhibiting clinical signs included in the differential diagnosis of HARD. Antigen tests were negative in all cats included in the study. The list of clinical signs observed in the cats of Group C can be seen in Table 1.

The average age of the cats in the study was 4.60 ± 3.36 years, with Group A having an average age of 4.64 ± 3.33 years, Group B 4.27 ± 3.66 years, and Group C 4.67 ± 3.40 years. No significant statistical differences were found in age among the groups. The average weight of the cats was 3.72 ± 1.11 kg (3.51 ± 0.56 kg for cats from Group A, 3.86 ± 1.03 kg for cats from Group B, and 3.89 ± 1.57 kg cats from Group C). There were no significant differences in weight between the groups. During the echocardiographic study, adult parasites were not found in any of the cats.

No quantitative abnormalities in the shape or tortuosity of the pulmonary vasculature were found on any of the radiographs studied. The obtained radiographic measurements are shown in Table 2. A VHS value for Group A was determined as 6.43 ± 0.92 (with an upper limit of 7.3), with no significant differences between the groups in terms of the VHS value. However, 37.21% (10/43) of cats from Group C showed cardiomegaly based on established reference values [25].

For the CVC/Ao and CVC/R4 ratios, the results from Group A showed a mean value of 1.09 ± 0.12 (upper limit 1.22) and 1.62 ± 0.15 (upper limit 1.77), respectively, being significantly higher in the seropositive cats for both ratios (Table 2). Moreover, statistically significant differences were present between Groups B and C (*p* = 0.003 for CVC/Ao and *p* = 0.021 for CVC/R4).

The mean value for the CrPA/R4 ratio for Group A was 0.76 ± 0.05 (upper limit 0.81). Statistically significant differences were observed between healthy cats and the rest of the studied groups (*p* < 0.001); however, no significant differences were observed between Groups B and C for this parameter (Table 2).

For ventrodorsal projection, the obtained ratio for CdPA/R9 was 0.79 ± 0.05 (upper limit 0.84). Statistically significant differences were observed between Group A and the rest of the studied groups (*p* < 0.001). Also, a statistically significant difference was presented between Group B and Group C (*p* = 0.017) (Table 2).

To determine if there is a correlation between the parameters with age and weight, the Pearson correlation model was used. Initially, the results showed a low positive or negative correlation between radiographic measurements and age or weight, and only a moderate positive correlation was observed between CdPA/R9 and weight. Finally, a moderate positive correlation was determined between the CVC and VHS (Table 3).

Regarding the obtained results for pulmonary patterns, Group A cats did not show any lung abnormalities. The results pertaining to cats in Groups B and C showed various lung parenchymal abnormalities including bronchial, mild and marked bronchointerstitial, and alveolar patterns (Table 4). The findings indicated that the predominant pulmonary pattern in Group B was a mild bronchointerstitial pattern (33.3%; 10/30), while in Group C, a marked bronchointerstitial pattern was observed in 62.8% (27/43) of cats. On the other hand, 30% (9/30) of cats in Group B showed no radiological abnormalities at the level of the lung parenchyma. A chi-squared test was performed to confirm the correlation between the presence/absence of clinical signs and the presence/absence of lung parenchymal abnormalities in cats from Groups B and C, irrespective of the type of radiological lung abnormality and its severity. A strong correlation was found between the presence of clinical signs and the presence of lung parenchymal abnormalities, with a statistically significant difference (*p* < 0.001) (Table 4).

## 4. Discussion

The diagnosis of heartworm infection in cats is far more complex than in dogs due to the specific characteristics of the feline host, such as a low parasite load [3,10]. In the case of juvenile or pre-adult worm infections, diagnosis is virtually impossible and therefore very complicated. Thus, the objective identification of compatible radiographic changes may be useful as an indicator of a high suspicion of HARD in infected cats. In infections with adult parasites, thoracic radiography is a valuable diagnostic and monitoring tool for the diagnosis of feline heartworm disease [26,31]. However, it has never been objectively assessed in cats with high suspicion of HARD. Radiographic abnormalities may be less consistent in feline heartworm disease than in canine heartworm disease, and the absence of such abnormalities does not exclude the diagnosis of heartworm disease in cats [3,12,32]. Therefore, this study was undertaken to evaluate whether specific and objective radiographic features of the heart and pulmonary vasculature could aid in the diagnosis of HARD.

The VHS values for healthy cats were similar to those previously reported by other authors (6.7–8.1, mean 7.5) [25,33]. The results indicated no significant differences in the VHS between the different groups, in contrast to what was reported by Litster et al. [26], who found that the mean VHS for the heartworm-infected cats was significantly greater than the reference value, and Venco et al. [17], who observed a tendency for the heart silhouette to increase in size during infection and at the onset of clinical signs. These differences may be attributed to the fact that the present study focused on cats with early infections, whereas the cited studies [17,26] were performed in cats with adult parasites. Nevertheless, it should be noted that 10 cats in Group C showed cardiomegaly, suggesting a trend towards an increased cardiac silhouette in cats with clinical signs, similar to that reported by Venco et al. [17]. However, the small variations in the analyzed parameters compared to healthy cats indicate the minimal usefulness of these measures as clinical diagnostic tools [17,26]. Indeed, the heart is rarely affected in feline heartworm disease [6].

A moderate (almost strong) positive correlation between the VHS and the diameter of the CVC was identified. Quite similar results (r = 0.59) were previously obtained in cats infected with *D. immitis* [26]; these authors reported that this finding, along with the increased mean VHS, may be linked to an elevation in filling pressures during the infection. Additionally, the CVC/Ao ratios were increased in infected cats compared to the healthy group; however, while the CVC/Ao ratio may provide some insights into right-sided heart conditions, pulmonary hypertension, and right ventricular hypertrophy, these are not typically observed in cats infected with *D. immitis* [6,10,15]. Similar findings were reported by Litster et al. [26], who found that the maximum width of the CVC in heartworm-infected dogs and cats was significantly greater than that obtained in the reference group, suggesting elevated right-sided heart filling pressures in both species. The authors argued that increased cardiac size and elevated filling pressures correlated and progressed together with heartworm disease. However, while the present study has shown an increased CVC/Ao ratio in infected cats, other potential factors influencing this measurement should be considered, and it should not be solely attributed to right-sided heart disease. Moreover, the echocardiographic exam carried out in the studied cats showed no evidence of right-sided heart disease. Further research is necessary to clarify the relationship between the CVC/Ao ratio and right-sided heart disease specifically in cats, given the unique aspects of feline heartworm infection.

Regarding the results obtained for the CVC/R4 ratio, similar results were observed, with the highest mean values seen in Group C. These findings are similar to those of other studies that examined the CVC/R4 ratio in dogs with heartworm with varying degrees of cardiac enlargement and found that this ratio increased with the severity of right ventricular enlargement [27,28]. The authors stated that, assuming that the degree of right ventricular enlargement was directly related to the severity and duration of heartworm infection, the relationship between CVC and right ventricular enlargement may reflect an increase in central venous pressure due to impending *cor pulmonale* [27]. This finding would be similar to that reported in cats, as discussed earlier [26], where it was suggested that increased cardiac size and elevated filling pressures occurred and were proportional to each other as the cardiac effects of heartworm disease progressed. Although the CCV/R4 ratio is not yet widely used in feline cardiology, these canine studies, along with the aforementioned study conducted in cats with heartworm, provide a reasonable basis for hypothesizing that an increased CCV/R4 ratio may indicate cardiac stress in cats with HARD, just as other authors have seen an increased VHS in cats with *D. immitis* showing clinical signs [17]. However, as mentioned earlier, further research is needed to determine the diagnostic utility of this ratio in feline heartworm disease.

The CrPA/R4 ratios obtained for healthy cats were very similar to those previously reported, which were 0.7 ± 0.13 [30]. The results showed that this ratio was significantly increased in cats with heartworm and, regardless of the presence or absence of clinical signs, these values were elevated in a large proportion of the animals infected with *D. immitis.* This differs from that reported by other authors, who subjectively reported that cranial lobar vessels were not enlarged in cats with heartworm [32]. However, previous studies found that cats infected with immature parasites had a significant increase in wall thickness, with occlusive medial hypertrophy present in 50% of cats infected by immature worms [34]; moreover, these authors argued that it was possible that medial hypertrophy of the small pulmonary arteries in exposed cats represented a pathologic response to transient heartworm infection. The first detectable pulmonary lesions of *D. immitis* infection include arteritis, pneumonitis, and hypertrophy of smooth muscle cells in the tunica media of small pulmonary arteries, which are likely attributable to pulmonary embolization of fifth-stage larvae before the establishment of infection with adult heartworms [35,36]. Therefore, it would be expected that certain arterial abnormalities would be found in the thoracic radiographs of these cats.

Similarly, the results showed values for CdPA/R9 ratios in healthy cats within the reference ranges usually considered (<1) [37]. Other authors have established significantly higher reference values for healthy cats (1.37 ± 0.28 for CdPA/R9) [32]. The reason for these differences beyond interobserver variability is unknown. Several authors agreed that a CdPA/R9 ratio greater than 1.6 has previously been reported in association with feline heartworm disease [26,32]. The results of the present study do not show such a pronounced thickening, likely because the cats examined were in the early stages of infections with immature parasites, while other studies involved chronic infections with adult parasites and, therefore, more advanced arterial damage [9]. However, the presence of significant differences between the seropositive cats and the Control Group is indicative of vascular lesions; moreover, these ratios were higher in cats with clinical signs. As noted above, in HARD, occlusive medial hypertrophy of the small pulmonary arterioles occurs, as well as changes in the pulmonary arteries [6]. Death of *D. immitis* in the pre-cardiac stages can also lead to smooth muscle hypertrophy of pulmonary arterioles.

In cats with HARD, changes are observed in the bronchi, bronchioles, and alveoli [5,6,23,34]. In this study, the lung patterns observed were more severe in feline patients with clinical signs, as lung parenchymal abnormalities were also observed in asymptomatic patients. Cats with clinical signs showed predominantly marked bronchointerstitial patterns, while asymptomatic cats showed milder forms. This is consistent with the pathophysiology of the feline heartworm and the pathogenesis of HARD, which is caused by the death of immature worms upon reaching the lungs. Obviously, this leads to clinical signs and radiological changes. Conversely, other studies have reported no correlation between radiographic lesions, clinical signs, or antibody levels [38]; however, this was a study based on a small number of cats (n = 10), all infected with adult worms. On the other hand, it has been described that infected cats may present with apparently normal thoracic radiographs [3,10], as observed in this study. The cardiopulmonary response to heartworm infection is dynamic and radiographs may not show changes if they are produced very early or very late in the course of the disease [11]. When present, feline heartworm should be considered in cats whose clinical and epidemiological characteristics are consistent with the infection.

A cat can remain seropositive for anti*-D.-immitis* antibodies for up to a year after clearance of the infection [23], so the seropositivity of the study cats does not necessarily indicate active infections, especially in asymptomatic cats, which is a limitation of this study. Moreover, the radiographic lesions of feline heartworm infection are dynamic over time, as demonstrated in experimental cat models where the timing of infection was known with accuracy [6,10,38]; as these cats were naturally infected, the exact time of infection is unknown, which may have affected the results. Nonetheless, these were infected cats that had been exposed to larval forms of the parasite in a hyperendemic region. In a study in which cats were experimentally inoculated with 100 L3 and subsequently treated with macrocyclic lactones as early as 70 days after infection, they showed radiographic and histopathological changes consistent with HARD at necropsy [5,23]. In addition, a large study with client-owned cats showed that 28% of heartworm-infected cats were asymptomatic [39].

## 5. Conclusions

The radiographic changes observed in the cats of this study indicated the presence of vascular and parenchymal abnormalities in those likely infected by immature *D. immitis* parasites. This was particularly evident in cats exhibiting clinical signs consistent with HARD, suggesting early vascular damage caused by this parasite. Given the challenges in diagnosing infections by immature *D. immitis* worms in cats, the examination of these radiographic measurements could serve as a valuable diagnostic tool for veterinary clinicians when HARD is strongly suspected. The results of the present study support the diagnostic suspicion of HARD in cats with compatible clinical signs and radiographic findings; however, heartworm infection should not be entirely ruled out in cats showing normal thoracic radiographs.

## Figures and Tables

**Table 1:** Summary of clinical signs observed in the study cats from Group C. Legend: n = number of cats showing the clinical sign described. Percentage (%) = percentage of cats in Group C showing the described clinical sign.

Clinical Sign	Number of Cats (n)	Percentage (%)
**Cough**	18	41.9%
**Tachypnea**	14	32.6%
**Respiratory distress**	23	53.5%
**Vomiting**	4	9.3%

**Table 2:** Results expressed by radiographic measurements and groups. Legend: VHS (Vertebral Heart Size); CrPA/R4 (right cranial pulmonary artery passing through the fourth rib in the laterolateral projection ratio); CVC/Ao (ratio of the caudal vena cava and diameter of the descending aorta in the laterolateral projection); CVC/R4 (caudal vena cava expressed as a ratio of the diameter of the fourth rib in the laterolateral projection); CdPA/R9 (right caudal pulmonary artery to the ninth rib in the dorsoventral projection ratio). Group A: seronegative asymptomatic cats; Group B: asymptomatic cats seropositive to anti-*Dirofilaria-immitis* antibodies; Group C: cats with clinical signs that were seropositive to *D. immitis*. Results are expressed as mean ± standard deviation.

Measure	Groups	Results	*p*-Value	R Effect	Interpretation
**VHS**	**Group A**	6.43 ± 0.92	0.27 ^ns^	0.05472692	No statistically significant differences between seronegative and seropositive cats
	**Group B**	5.98 ± 1.04	0.41655099 ^ns^	0.02500844	No statistically significant differences between Groups B and C
	**Group C**	6.61 ± 1.62			
**CrPA/R4**	**Group A**	0.76 ± 0.05	3.0545 × 10^−21^ **	0.74419009	Statistically significant difference between seronegative and seropositive cats
	**Group B**	1.12 ± 0.22	0.41783262 ^ns^	0.02427917	No statistically significant differences between Groups B and C
	**Group C**	1.17 ± 0.19			
**CVC/Ao**	**Group A**	1.09 ± 0.12	5.02886 × 10^−7^ **	0.44459144	Statistically significant difference between seronegative and seropositive cats
	**Group B**	1.16 ± 0.14	0.00349851 **	0.31565838	Statistically significant difference between Groups B and C
	**Group C**	1.35 ± 0.25			
**CVC/R4**	**Group A**	1.62 ± 0.15	1.2276 × 10^−21^ **	0.74896233	Statistically significant difference between seronegative and seropositive cats
	**Group B**	2.41 ± 0.61	0.021834542 *	0.23490465	Statistically significant difference between Groups B and C
	**Group C**	2.62 ± 0.47			
**CdPA/R9**	**Group A**	0.79 ± 0.05	8.7566 × 10^−22^ **	0.75130749	Statistically significant difference between seronegative and seropositive cats
	**Group B**	1.23 ± 0.31	0.017873607 *	0.24919937	Statistically significant difference between Groups B and C
	**Group C**	1.36 ± 0.34			

**, Correlation is significant at 0.5% (*p* < 0.005); *, correlation is significant at 5% level (*p* < 0.05); ns, correlation is not significant.

**Table 3:** Correlation coefficient for all studied parameters with weight and age values, as well as correlation between CVC and VHS.

Correlation	Coefficient	Interpretation
**VHS–Weight**	0.26410673 **	Low positive correlation
**VHS–Age**	0.2625266 *	Low positive correlation
**CrPA/R4–Weight**	0.24220781 *	Low positive correlation
**CrPA/R4–Age**	−0.02621345 ^ns^	Low negative correlation
**CVV/Ao–Weight**	0.27288863 **	Low positive correlation
**CVV/Ao–Age**	−0.06015906 ^ns^	Low negative correlation
**CVV/R4–Weight**	−0.0537349	Low negative correlation
**CVV/R4–Age**	−0.1179146 ^ns^	Low negative correlation
**CdPA/R9–Weight**	0.41493029 **	Moderate positive correlation
**CdPA/R9–Age**	0.0134005 ^ns^	Low positive correlation
**VCC–VHS**	0.4903414 **	Moderate positive correlation

**, Correlation is significant at 0.5% (*p* < 0.005); *, correlation is significant at 1% level (*p* < 0.01); ns, correlation is not significant.

**Table 4:** Lung parenchymal abnormalities observed in the studied cats. Group B: asymptomatic cats seropositive to anti-*Dirofilaria-immitis* antibodies; Group C: cats with clinical signs that were seropositive to *D. immitis*. Results are expressed as percentage (%) as well as number of cats exhibiting each lung pattern by group.

Lung Parenchymal Abnormalities	Group B (n = 30)	Group C (n = 43)	Groups B + C (n = 73)
**Bronchial pattern**	20% (6/30)	4.6% (2/43)	10.9% (8/73)
**Bronchointerstitial pattern (mild)**	33.3% (10/33)	13.9% (6/43)	21.9% (16/73)
**Bronchointerstitial pattern (marked)**	16.7% (5/30)	62.8% (27/43)	43.8% (32/73)
**Alveolar + interstitial pattern**	0% (0/30)	18.6% (8/43)	10.9% (8/73)
**Total**	70% (21/30)	100% (43/43)	87.6% (64/73)

## Data Availability

All data generated or analyzed during this study are included in this article. The datasets used and/or analyzed during the present study are available from the corresponding author upon reasonable request.
